# A single-blind, dose escalation, phase I study of high-fluence light-emitting diode-red light (LED-RL) on human skin: study protocol for a randomized controlled trial

**DOI:** 10.1186/s13063-016-1518-7

**Published:** 2016-08-02

**Authors:** Derek Ho, Ekaterina Kraeva, Ted Wun, R. Rivkah Isseroff, Jared Jagdeo

**Affiliations:** 1 Dermatology Service, Sacramento VA Medical Center, Mather, CA USA; 2 Department of Dermatology, University of California Davis, Sacramento, CA USA; 3 Division of Hematology Oncology, Department of Internal Medicine, University of California Davis, Sacramento, CA USA; 4 Division of Hematology Oncology, VA Northern California Healthcare System, Mather, CA USA; 5 UC Davis Clinical and Translational Sciences Center, Sacramento, CA USA; 6 Department of Dermatology, State University of New York Downstate Medical Center, Brooklyn, NY USA

**Keywords:** Light-emitting diode-red light, LED-RL, High-fluence, Phototherapy, Skin fibrosis, Wound healing, Scar, Keloid, RCT

## Abstract

**Background:**

Skin fibrosis is involved in a variety of pathologic conditions ranging from scar formation secondary to surgery or trauma to immune-mediated processes. Skin fibrosis is a significant international health problem with an estimated incidence of greater than 100 million people affected per year worldwide with few effective treatment options available. Preliminary in vitro data generated by our research group suggests that red light can function as a stand-alone treatment for skin fibrosis. To our knowledge, no prior clinical trials have been performed to determine the safety of high-fluence (dose) light-emitting diode-red light (LED-RL) phototherapy. The goal of this study is to evaluate the safety of LED-RL fluences from 160 J/cm^2^ up to 640 J/cm^2^ in healthy subjects.

**Methods/design:**

This is a single-blind, dose escalation, randomized controlled, phase I study to evaluate the safety of high-fluence LED-RL on human skin. The protocol for dose escalation requires subjects be enrolled sequentially in groups of five. Within each group, three subjects will be randomized to LED-RL phototherapy and two subjects randomized to mock therapy. Subjects in group 1 randomized to LED-RL phototherapy will receive the maximum recommended starting dose (160 J/cm^2^). LED-RL dose will be escalated in subsequent groups (320 J/cm^2^, 480 J/cm^2^ and 640 J/cm^2^). The maximally tolerated dose (MTD) is defined as the dose level below the dose producing unacceptable but reversible toxicity and is considered to be the upper limit of subject tolerance. After either a MTD has been established, or the study endpoint of 640 J/cm^2^ has been achieved, an additional 27 LED-RL phototherapy subjects (for a total of 30) and 18 mock therapy subjects (for a total of 20) (determined randomly) will be enrolled. Each subject will receive a total of nine procedures, three times per week for three consecutive weeks.

**Discussion:**

This study may provide important safety information on the effects of high-fluence LED-RL phototherapy on human skin and help facilitate future phase II studies to evaluate the efficacy of high-fluence LED-RL as a potential noninvasive, safe, portable, at-home therapy for treatment of skin fibrosis.

**Trial registration:**

ClinicalTrials.gov NCT02630303. Registered on 9 December 2015.

**Electronic supplementary material:**

The online version of this article (doi:10.1186/s13063-016-1518-7) contains supplementary material, which is available to authorized users.

## Background

Skin fibrosis is involved in a variety of pathologic conditions ranging from scar formation secondary to surgery or trauma, such as hypertrophic or keloid scars, to immune-mediated processes, such as scleroderma and chronic graft-versus-host disease. Skin fibrosis is a significant international health problem with an estimated incidence of greater than 100 million people affected per year worldwide with few effective treatment options available [1]. In addition, skin fibrosis is a significant socioeconomic burden due to the functional, aesthetic, and psychosocial impacts on patients’ lives [1, 2]. A consumer survey of the American Society for Dermatologic Surgery (ASDS) reported that over 50 % of survey respondents considered treatment with laser and light therapy to improve scar appearance and skin discoloration [3]. In 2014, members of the ASDS performed more than 2.7 million procedures using lasers, lights, and energy-based devices [4]. Lasers and light-based devices have gained increased attention and their use has expanded amongst dermatologists and patients alike due to minimal downtime associated with phototherapy procedures and convenient at-home use of many devices.

Many of the currently available treatments for skin fibrosis have certain pitfalls and limitations. For example, immunosuppressive agents such as systemic corticosteroids may lead to serious systemic side effects. Anti-fibrotic agents such as intralesional steroids, 5-fluorouracil, and bleomycin are invasive, painful, and have associated skin and systemic effects [5–9]. Ultraviolet (UVA/UVA1) and UVB/narrowband UVB phototherapy generate UV-induced deoxyribonucleic acid (DNA) damage that is associated with skin cancer and/or photoaging [10].

Recently published clinical observations indicate that red light (600 to 700 nm) in combination with other modalities such as photosensitizers in combined red light photodynamic therapy can lessen skin fibrosis [11–13]. Red light has a penetration depth of up to 4 mm, which allows it to reach the entirety of the dermis where skin fibrosis occurs [14, 15]. These findings make red light a promising treatment modality that is noninvasive, unlikely to cause systemic side effects, and does not generate pro-carcinogenic DNA damage as may occur in UV light treatment.

Preliminary in vitro data generated by our research group suggests that red light may function as a stand-alone treatment for skin fibrosis, eliminating the side effects of chemical photosensitizers [16, 17]. Furthermore, commercially available light-emitting diode-red light (LED-RL) units exist and are already Food and Drug Administration (FDA)-cleared for other dermatological uses (such as for improvement of rhytides and acne) [18, 19]. Thus, clinical translation for the use of LED-RL phototherapy in skin fibrosis could occur relatively quickly following demonstration of its safety and efficacy. Developing LED-RL phototherapy as a treatment modality would represent an important advance in therapy for scarring conditions, without the side effects associated with current treatment options.

To our knowledge, no prior clinical trials have been performed to determine the safety of high-fluence (dose) LED-RL phototherapy. High-fluence LED-RL phototherapy is phototherapy with fluence >160 J/cm^2^ per treatment session. The goal of this study is to establish the safety of LED-RL fluences from 160 J/cm^2^ up to 640 J/cm^2^ in healthy subjects, and to help facilitate future phase II efficacy studies of high-fluence LED-RL phototherapy as an innovative, safe, and efficacious treatment modality.

## Methods/design

### Hypothesis

High-fluence LED-RL phototherapy is safe on human skin.

### Study endpoints

This study will end if the maximally tolerated dose (MTD) is identified or if the predefined study endpoint of 640 J/cm^2^ is reached.

### Study design and subject population

This is a single-blind, dose escalation, randomized controlled, phase I study to evaluate the safety of high-fluence LED-RL phototherapy on human skin. The maximum recommended starting dose (MRSD) of 160 J/cm^2^ is based upon previously published maximum doses of LED-RL phototherapy used in clinical studies that demonstrated clinical safety with no adverse events [18, 19]. The study endpoint of 640 J/cm^2^ was chosen due to feasibility, as this dose corresponds to 2 hours of LED-RL phototherapy, and we anticipate decreased subject compliance with LED-RL exposures longer than 2 hours in duration. Additionally in prior studies, the 640 J/cm^2^ dose demonstrated increased anti-fibrotic properties in vitro compared to lower doses of LED-RL [17].

The protocol for dose escalation requires subjects to be enrolled sequentially in groups of five (three subjects randomized to LED-RL phototherapy and two subjects randomized to mock therapy). As this is a single-blind study, subjects will be blinded to the procedure (LED-RL phototherapy or mock therapy).

Subjects in group 1 randomized to LED-RL phototherapy will receive the MSRD of 160 J/cm^2^. The LED-RL dose will be escalated in subsequent groups using the classical method for dose escalation as described by Spilker: starting with dose (X) increased by an equal amount (in this instance: X = 160 J/cm^2^, 2X = 320 J/cm^2^, 3X = 480 J/cm^2^, and 4X = 640 J/cm^2^) [20]. Common expected procedural side effects are mild and are expected to last less than 24 hours, which include warmth, redness (erythema), and swelling (edema). The MTD is defined as the dose level below the dose producing unacceptable but reversible toxicity and is considered to be the upper limit of subject tolerance. Unacceptable but reversible toxicities (adverse events) for this study include: second-degree or higher skin burning or blistering, erythema lasting more than 24 hours, severe swelling, pain, ulceration, change in sensation, and/or muscle weakness. If one or more subject experiences any of these adverse events, then this is the dose one above the MTD, and we will not proceed with this dose or escalate the dose.

After either an MTD has been established, or the study endpoint of 640 J/cm^2^ has been achieved, an additional 27 LED-RL phototherapy subjects (for a total of 30) and 18 mock therapy subjects (for a total of 20) (determined randomly) will be enrolled to satisfy Hanley’s Rule of Three, such that it can be concluded with 95 % confidence that fewer than 1 person in 10 will experience an adverse event [21]. Of the larger cohort, the study will be stopped if adverse events determined to be device-related equal or exceed 30 %. Each subject will receive a total of nine LED-RL phototherapy or mock therapy procedures, three times per week for three consecutive weeks. The phototherapy regimen of three procedures per week is a standard phototherapy protocol [22]. Each subject will receive a US$50 check for completion of 1 week (total of US$150 for completion of entire study), and subjects will receive a prorated amount in the event of withdrawal from study.

This study has a sample size of up to 65 subjects. Subjects will be recruited from the Sacramento Veterans Affairs (VA) Medical Center. As detailed in the inclusion criteria, subjects may be of any sex, age, and ethnicity. The Standard Protocol Items: Recommendations for Interventional Trials (SPIRIT) checklist, flow diagram, and trial registration data are available as Additional files 1, 2, and 3.

### Inclusion and exclusion criteria

Please refer to Table 1 for a listing of study inclusion and exclusion criteria. All subjects will be tested for photosensitivity as per the manufacturer’s user guide instructions. A 20-minute session of LED-RL phototherapy will be performed on the subject’s nondominant proximal anterior forearm, and evaluation for photosensitivity will occur 24 hours post LED-RL phototherapy. Criteria for photosensitivity include but are not limited to: warmth, erythema (redness), edema (swelling), rash, pain, or discomfort lasting more than 24 hours.

**Table 1:** Study inclusion and exclusion criteria

Inclusion criteria	Exclusion criteria
• Healthy subjects of any sex, age, and ethnicity • Nondominant proximal anterior forearm is wide enough to ensure reproducible placement of light-emitting diode-red light (LED-RL) phototherapy or mock therapy hand-held unit • Available and willing to attend all clinic visits • Able and willing to give informed consent	• Subjects on any photosensitizing medications (i.e. lithium, melatonin, phenothiazine antipsychotics, antibiotics) • Subjects with light-sensitive conditions • Subjects with diabetes mellitus (DM) • Subjects with a history of skin cancer: basal cell carcinoma (BCC), squamous cell carcinoma (SCC), or melanoma • Subjects with systemic lupus erythematosus (SLE) • Subjects with any other medical condition that could be compromised by exposure to the proposed procedure • Subjects with open wounds on the nondominant proximal anterior forearm • Subjects with fibrotic skin disease or other skin conditions on the nondominant proximal anterior forearm • Subjects with tattoos that cover the procedure site on the nondominant proximal anterior forearm

### Specifications of LED-RL phototherapy and mock therapy hand-held unit

The LED-RL light source is a commercially available Omnilux New-U hand-held LED-RL phototherapy unit (provided by Photo Therapeutics, Carlsbad, CA, USA), and is FDA-cleared for treatment of periorbital rhytides (crow’s feet) and may be placed in direct contact with the skin [23]. The mock therapy unit, also provided by Photo Therapeutics, Carlsbad, CA, USA, only generates warmth and does not emit LED-RL. The LED has a 4.7 cm × 6.1 cm rectangular aperture and emits visible red light (633 nm ± 30 nm) at a power density of 360.2 W/m^2^ at room temperature in direct contact with the photometer.

### Study procedure

The study procedure for subjects receiving LED-RL phototherapy and for subjects receiving mock therapy is identical with the exception of utilizing the LED-RL phototherapy unit or the mock therapy unit for the randomized subjects in their respective groups. After confirming subject identity, screening for study eligibility, and obtaining written Institutional Review Board (IRB)-approved informed consent, the subject will be taken to a private clinic examination room at the Dermatology Clinic in Sacramento VA Medical Center. The subject’s nondominant proximal anterior forearm will be cleaned with alcohol pads. A surgical marking pen will be used to outline the procedure area at the start and completion of every visit. The LED-RL phototherapy or mock therapy hand-held unit will be held in place and in direct contact with the clean area using nonadhesive tape (ACE elastic bandage or similar) during the procedure. All subjects will be provided with protective eyewear to use at LED-RL phototherapy and mock therapy sessions. The research team will be observing the procedure and assessing for any safety issues during and immediately post-procedure. Photographs will be taken pre-and post-procedure at each clinic visit to record and assess for common expected procedural side effects and adverse events, and to ensure uniformity of procedure location at every clinic visit.

### Safety assessment

All subjects will be asked to record any common expected procedural side effects and adverse events in a subject diary of adverse events for the entire duration of study participation. A review of the subject’s diary will occur at each clinic visit prior to the start of the procedure. Subjects will be called weekly to monitor for any adverse events. Subjects with adverse events will receive standard medical care outside of the scope of this study. Ancillary and post-study care is available at the General Dermatology Clinic, Sacramento VA Medical Center, but no additional compensation will be provided to those who suffer harm from study participation. All common expected procedural side effects and adverse events will be described in the Case Report Form. The Data Safety Monitoring Board, which includes three board-certified dermatologists, one phototherapy nurse, and two clinical research coordinators, will convene monthly to review and assess any study safety issues. The principal investigator (PI: JJ) has access to interim results and may make the decision to stop a subject from study participation due to safety reasons.

### Randomization

**Fig. 1:**
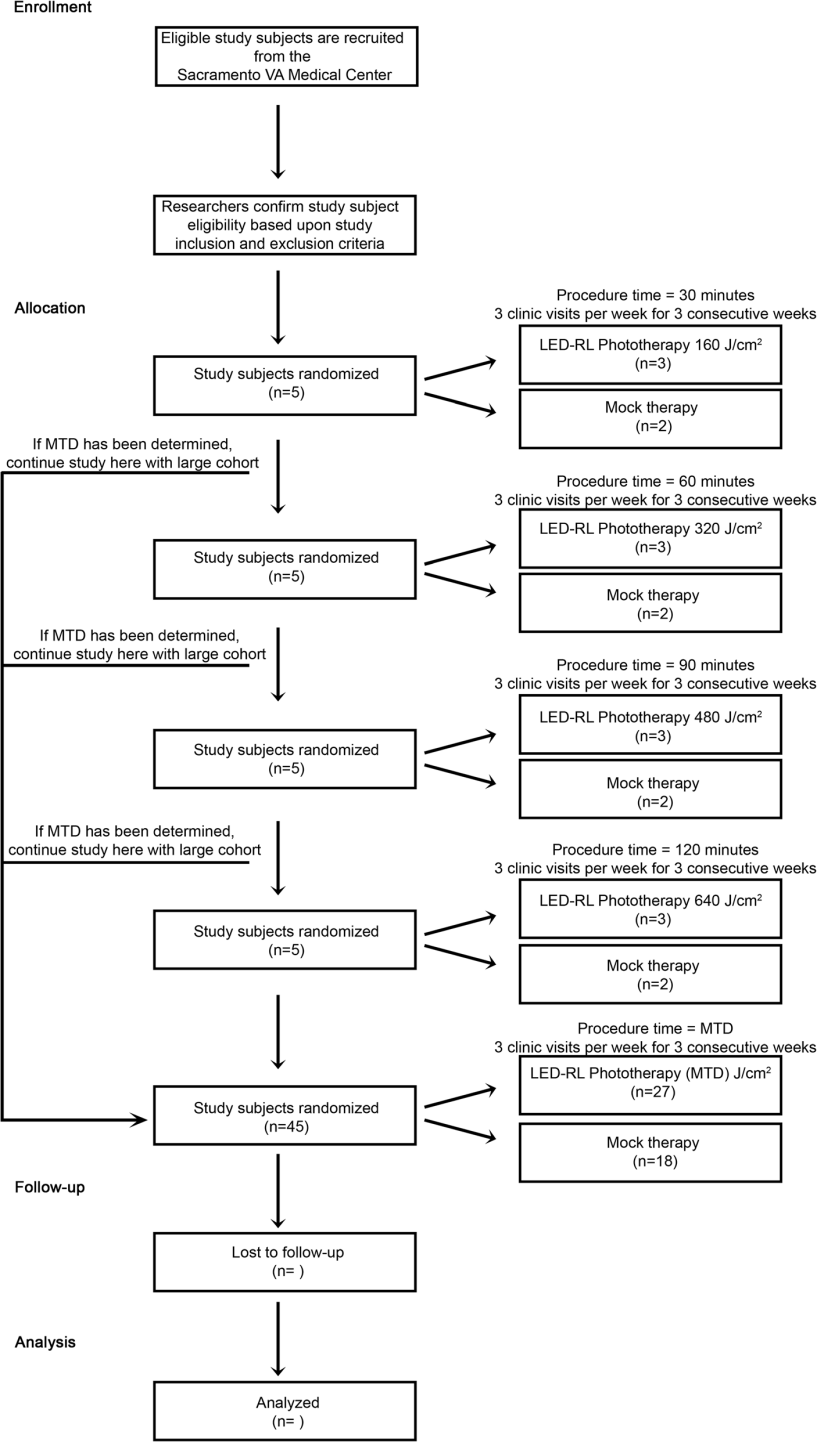
Study randomization flowchart. The maximally tolerated dose (MTD) is defined as the dose level below the dose producing unacceptable but reversible toxicity and is considered the upper limit of subject tolerance. Unacceptable but reversible toxicities (adverse events) for this study include: second-degree or higher skin burning or blistering, erythema lasting more than 24 hours, severe swelling, pain, ulceration, change in sensation, and/or muscle weakness

Randomization will be performed by clinical research coordinators via the www.randomizer.org website. For assignment of study groups, Arabic numerals “1,” “2,” “3,” “4,” and “5” will be generated at random. Subjects assigned to “1” will be in group 1 (160 J/cm^2^), “2” will be in group 2 (320 J/cm^2^), “3” will be in group 3 (480 J/cm^2^), “4” will be in group 4 (640 J/cm^2^), and “5” will be in group 5 (640 J/cm^2^ or MTD). For assignment of LED-RL phototherapy and mock therapy, Arabic numerals “1” and “2” will be generated at random. Subjects assigned to “1” will receive LED-RL phototherapy and subjects assigned to “2” will receive mock therapy. A study randomization flowchart is shown in Fig. 1.

### Blinding

This is a single-blind study, and the subjects will be blinded to the procedure (LED-RL phototherapy or mock therapy). The PI and clinical research coordinators (DH and EK) will be aware of the randomization.

### Time frame

This study is designed to conclude in 7 months, which includes subject recruitment, performing procedures with LED-RL phototherapy and mock therapy, and data analysis.

### Statistical analysis

Statistical analysis will be performed using Statistical Package for Social Sciences (SPSS) or similar. Quantitative data will be presented as mean ± standard deviation and range while qualitative data will be presented as number (*n*) and percentage (%). Student’s *t* test will be used to determine if there is a significant difference in the rate of common expected procedural side effects and adverse events between the LED-RL phototherapy group and the mock therapy group. *P* <0.05 will be used as statistically significant. Data analysis relating to protocol nonadherence and any missing data will be consulted with the biostatistics service at UC Davis Clinical and Translational Science Center.

## Discussion

To our knowledge, no prior clinical trials have been performed to determine the safety of high-fluence LED-RL phototherapy. This study may provide important safety information on high-fluence LED-RL phototherapy (160 J/cm^2^ up to 640 J/cm^2^) and help facilitate future phase II studies to evaluate the efficacy of high-fluence LED-RL as a potential noninvasive, safe, portable, at-home therapy for treatment of skin fibrosis. In addition, future phase II clinical trials may reference this study to determine an optimal dose of LED-RL phototherapy for treatment of skin fibrosis.

There may be several potential limitations of this study, which may be encountered at the Sacramento VA Medical Center, Mather, CA, USA. There may be bias towards the male sex in a veteran population, with women representing only 9 % of veterans nationwide [24]. Men have more skin collagen and thicker skin in comparison to women [25], and thus may require higher doses of LED-RL to produce an adverse event. Thus, the MTD of LED-RL phototherapy may differ between men and women. In addition, there may be bias in age toward middle-aged and elderly subjects recruited within the veteran population. Since increased age is associated with reduced skin collagen [26], the penetration of LED-RL may be affected, and the total dose that results in an adverse event may vary amongst different age groups. Consequently, the MTD obtained from this study may only be representative for the sampled age groups. Furthermore, there is bias in ethnicity toward Caucasians within the veteran population, with the minority of veterans nationwide being represented by approximately 12 % African Americans, 7 % Hispanics, and 4 % other ethnicities [24]. The research team will attempt to minimize bias by approaching and recruiting subjects of any sex, age, and ethnicity.

To our knowledge, this is the first phase I clinical trial investigating high-fluence LED-RL on human skin. Therefore, we are conducting a single-site clinical trial. This phase I study was designed with consultation from the Clinical and Translational Science Center (CTSC) Biostatistics and Bioethical services. Randomization and recruitment of a variety of subjects will help minimize bias. Limited data exists pertaining to LED-RL safety and effect in different Fitzpatrick skin types or pigmentation. Post-phase I trial statistical analysis will determine differential safety of LED-RL associated with race and ethnicity and examine to see whether this should be further pursued using more subjects with greater power in a future study, or dose stratification based upon pigmentation. Future multicenter phase I studies stratifying race and ethnicity can be done to determine whether different skin types have different MTDs.

Future phase II studies may evaluate the efficacy of high-fluence LED-RL phototherapy as a potential treatment modality for skin fibrosis based upon the safety information obtained from this study. Following successful demonstration of the safety of high-fluence LED-RL phototherapy on human skin, our research group may pursue a phase II split-scar study to evaluate the efficacy of high-fluence LED-RL phototherapy. Successful demonstration of the efficacy of high-fluence LED-RL phototherapy for treatment of skin fibrosis has the potential to benefit many individuals worldwide, as high-fluence LED-RL phototherapy may lack the side effects that are associated with currently available treatment options.

### Study status

This study began enrolling subjects in January 2016 at the Sacramento VA Medical Center, Mather, CA, USA.

## Abbreviations

ASDS, American Society for Dermatologic Surgery; IRB, Institutional Review Board; LED-RL, light-emitting diode-red light; MSRD, maximum recommended starting dose; MTD, maximum tolerated dose; UV, ultraviolet; VA, Veterans Affairs

## Additional files

Additional file 1: SPIRIT checklist. (PDF 502 kb) Additional file 2: SPIRIT flow diagram. (DOC 50 kb) Additional file 3: SPIRIT trial registration data. (DOCX 110 kb)
