# Supplementary material for: A single-blind, dose escalation, phase I study of high-fluence light-emitting diode-red light (LED-RL) on human skin: study protocol for a randomized controlled trial
Source: Trials. 2016 Aug 2;17:385. doi: 10.1186/s13063-016-1518-7 (PMC4971661; doi:10.1186/s13063-016-1518-7)
Supplement: Additional file 2: — SPIRIT flow diagram. (DOC 50 kb) [file 13063_2016_1518_MOESM2_ESM.doc]

SPIRIT Figure. Time schedule of enrollment, interventions, and assessments.

|  | **STUDY PERIOD** | | | | | | | |
| --- | --- | --- | --- | --- | --- | --- | --- | --- |
|  | **Enrollment** | **Allocation** | **Post-allocation** | | | | | **Close-out** |
| **TIMEPOINT** | **Month 1-5** | **Month 1-5** | **Month 1** | **Month 2** | **Month 3** | **Month 4** | **Month 5** | **Month**  **6-7** |
| **ENROLLMENT:** |  | | | | | | | |
| **Eligibility screen** | X |  |  |  |  |  |  |  |
| **Informed consent** | X |  |  |  |  |  |  |  |
| **Photosensitivity evaluation** | X |  |  |  |  |  |  |  |
| **Allocation: Randomized (LED-RL or mock procedure)** |  | X |  |  |  |  |  |  |
| **INTERVENTIONS:** |  | | | | | | | |
| **160 J/cm^2^** |  |  | X |  |  |  |  |  |
| **320 J/cm^2^** |  |  |  | X |  |  |  |  |
| **480 J/cm^2^** |  |  |  |  | X |  |  |  |
| **640 J/cm^2^** |  |  |  |  |  | X |  |  |
| **MTD (large cohort)** |  |  |  |  |  |  | X |  |
| **ASSESSMENTS:** |  | | | | | | | |
| **Safety** | X |  | X | X | X | X | X |  |
| **Data Analysis/**  **Manuscript Preparation** |  |  |  |  |  |  |  | X |
