# Supplementary material for: A single-blind, dose escalation, phase I study of high-fluence light-emitting diode-red light (LED-RL) on human skin: study protocol for a randomized controlled trial
Source: Trials. 2016 Aug 2;17:385. doi: 10.1186/s13063-016-1518-7 (PMC4971661; doi:10.1186/s13063-016-1518-7)
Supplement: Additional file 3: — SPIRIT trial registration data. (DOCX 110 kb) [file 13063_2016_1518_MOESM3_ESM.docx]

**Trial Registration Data**

| **Data category** | **Information** |
| --- | --- |
| Primary registry and trial identifying number | ClinicalTrials.gov NCT02630303 |
| Date of registration in primary registry | December 9, 2015 |
| Secondary identifying numbers | 15-12-00756 |
| Source(s) of monetary or material support | None |
| Primary sponsor | Jared Jagdeo, MD, MS |
| Secondary sponsor(s) | None |
| Contact for public queries | Jared Jagdeo, MD, MS [jrjagdeo@gmail.com] |
| Contact for scientific queries | Jared Jagdeo, MD, MS [jrjagdeo@gmail.com] |
| Public title | High Fluence Light Emitting Diode-Red Light (LED-RL) in Human Skin |
| Scientific title | Phase I study of high fluence light emitting diode-red light (LED-RL) in human skin: A single-blind, dose escalation, randomized controlled trial |
| Countries of recruitment | United States of America |
| Health condition(s) or problem(s) studied | Skin fibrosis, scar, keloid |
| Intervention(s) | LED-RL: dose of 160, 320, 480, and 640 J/cm^2^  Mock: mock procedure |
| Key inclusion and exclusion criteria | Inclusion Criteria:   - Healthy subjects of any sex, ethnicity and age - Nondominant proximal anterior forearm is wide enough to ensure reproducible placement of LED-RL phototherapy or mock therapy hand-held unit - Available and willing to attend all clinic visits - Able and willing to give informed consent   Exclusion Criteria:   - Subjects using any photosensitizers (i.e. lithium, melatonin, phenothiazine antipsychotics, antibiotics) - Subjects with diabetes mellitus (DM) - Subjects with a history of skin cancer; basal cell carcinoma (BCC) or squamous cell carcinoma (SCC). - Subjects with systemic lupus erythematous (SLE) - Subjects with any other medical condition that could be compromised by exposure to the proposed treatment - Subjects with light-sensitive conditions or on photosensitizing medications (All subjects will be tested for photosensitivity per manufacturer user guide instructions) - Subjects with open wounds on the nondominant proximal anterior forearm - Subjects with fibrotic skin disease or other skin conditions on the nondominant proximal anterior forearm - Subjects with tattoos that cover the procedure site on the nondominant proximal anterior forearm |
| Study type | Interventional  Allocation: randomized; Intervention model: parallel assignment; Masking: single-blind  Primary purpose: safety evaluation  Phase I |
| Date of first enrollment | January 2016 |
| Target sample size | 65 |
| Recruitment status | Recruiting |
| Primary outcome(s) | To evaluate safety of high fluence LED-RL in human skin |
| Key secondary outcomes | None |
